# A Highly Conserved Residue in HIV-1 Nef Alpha Helix 2 Modulates Protein Expression

**DOI:** 10.1128/mSphere.00288-16

**Published:** 2016-11-09

**Authors:** Aaron L. Johnson, Brennan S. Dirk, Mathieu Coutu, S. M. Mansour Haeryfar, Eric J. Arts, Andrés Finzi, Jimmy D. Dikeakos

**Affiliations:** a Department of Microbiology and Immunology, Schulich School of Medicine and Dentistry, University of Western Ontario, London, Ontario, Canada; b Centre de Recherche du CHUM, Department of Microbiology and Immunology, Infection and Immunology, Université de Montréal, Montréal, Québec, Canada; University of Pittsburgh School of Medicine

**Keywords:** Nef, diversity, human immunodeficiency virus

## Abstract

Extensive genetic diversity is a defining characteristic of human immunodeficiency virus type 1 (HIV-1) and poses a significant barrier to the development of an effective vaccine. To better understand the impact of this genetic diversity on the HIV-1 pathogenic factor Nef, we compiled a panel of reference strains from the NIH Los Alamos HIV Database. Initial sequence analysis identified point mutations at Nef residues 13, 84, and 92 in subtype C reference strain C.BR92025 from Brazil. Functional analysis revealed impaired major histocompatibility complex class I and CD4 downregulation of strain C.BR92025 Nef, which corresponded to decreased protein expression. Metabolic labeling demonstrated that strain C.BR92025 Nef has a greater rate of protein turnover than subtype B reference strain B.JRFL that, on the basis of mutational analysis, is related to Nef residue A84. An alanine-to-valine substitution at position 84, located in alpha helix 2 of Nef, was sufficient to alter the rate of turnover of an otherwise highly expressed Nef protein. In conclusion, these findings highlight HIV-1 Nef residue A84 as a major determinant of protein expression that may offer an additional avenue to disrupt or mediate the effects of this key HIV-1 pathogenic factor.

**IMPORTANCE** The HIV-1 Nef protein has been established as a key pathogenic determinant of HIV/AIDS, but there is little knowledge of how the extensive genetic diversity of HIV-1 affects Nef function. Upon compiling a set of subtype-specific reference strains, we identified a subtype C reference strain, C.BR92025, that contained natural polymorphisms at otherwise highly conserved residues 13, 84, and 92. Interestingly, strain C.BR92025 Nef displayed impaired Nef function and had decreased protein expression. We have demonstrated that strain C.BR92025 Nef has a higher rate of protein turnover than highly expressed Nef proteins and that this higher rate of protein turnover is due to an alanine-to-valine substitution at Nef residue 84. These findings highlight residue A84 as a major determinant of HIV-1 Nef expression.

## Abstract (precis)

The HIV-1 Nef protein has been established as a key pathogenic determinant of HIV/AIDS, but there is little knowledge of how the extensive genetic diversity of HIV-1 affects Nef function. Upon compiling a set of subtype-specific reference strains, we identified a subtype C reference strain, C.BR92025, that contained natural polymorphisms at otherwise highly conserved residues 13, 84, and 92. Interestingly, strain C.BR92025 Nef displayed impaired Nef function and had decreased protein expression. We have demonstrated that strain C.BR92025 Nef has a higher rate of protein turnover than highly expressed Nef proteins and that this higher rate of protein turnover is due to an alanine-to-valine substitution at Nef residue 84. These findings highlight residue A84 as a major determinant of HIV-1 Nef expression.

## INTRODUCTION

In addition to the structural and functional proteins that are essential for viral replication and virion production, human immunodeficiency virus type 1 (HIV-1) encodes a number of accessory proteins that are required for optimal *in vivo* replication (1, 2). Among these accessory proteins is the 27-kDa N-myristoylated protein Nef. Originally referred to as the “negative factor,” because of early reports that erroneously claimed a role in negative regulation of viral replication (3), Nef has since been established as a major pathogenic determinant of AIDS (4).

Analysis of HIV-1-infected individuals displaying dramatically decreased progression to AIDS revealed gross defects in the *nef* gene (4–8). Additionally, rhesus macaques infected with a Nef-deficient simian immunodeficiency virus (SIV) have significantly better survival outcomes than those infected with SIV producing a functional Nef protein (1). Moreover, a transgenic mouse model expressing Nef from the CD4 promoter demonstrated that Nef expression alone was sufficient to cause an AIDS-like phenotype in mice (9).

The prominent role HIV-1 Nef plays in disease progression is striking given the apparent lack of enzymatic activity (10). However, Nef is a multifunctional protein capable of interacting with many cellular host proteins (11). Such interactions enable Nef functions in altering T cell activation (12, 13), increasing virion infectivity (14, 15), and modulating membrane trafficking to downregulate cell surface receptors (16), among others. The best-studied functions of Nef are the downregulation of major histocompatibility complex class I (MHC-I) (17–20) and CD4 (21). Downregulation of MHC-I by Nef prevents the detection of virus-infected cells by cytotoxic T lymphocytes (CTLs) (22), whereas removal of CD4 from the cell surface by Nef limits the killing of infected CD4^+^ T cells by antibody-dependent cell-mediated cytotoxicity (23, 24) and unfavorable superinfection, thereby increasing viral dissemination (25).

Despite vast improvements in our ability to prevent, detect, and treat HIV/AIDS, this chronic disease remains a major global health concern, with over 36 million infected individuals globally by the end of 2015 (26, 27). One of the reasons a vaccine and/or cure for HIV-1 has been so elusive is the extensive genetic diversity of the virus (28, 29). Specifically, HIV-1 is divided into four groups (M, N, O, and P) with <70% nucleotide sequence homology (30). These groups can be further subdivided into 10 subtypes (A through K) (31) that differ up to 10 to 15% in amino acid diversity in the viral proteins encoded at the 3′ end of the HIV-1 genome (i.e., Vpu, Tat, rev, Env, and Nef) (30). Whereas HIV-1 subtype B is responsible for approximately 10% of the epidemic (~3 to 4 million cases), subtypes A, C, and D, as well as recombinants of these subtypes, are dominant around the world and responsible for over 30 million infections (32). Early HIV-1 research focused mainly on subtype B, which is most prevalent in North America and Western Europe (33, 34), and as a result, there remains a gap in our understanding of how this genetic diversity affects HIV-1 biology.

Overall, amino acid residues of high entropy/low conservation in the HIV-1 proteome often map to regions under constant selective pressure in part because of differential immune responses and restrictive factors between human hosts (30). Regions of higher conservation often predict viral protein sequences of structural and functional importance. However, in HIV-1, very few positions in the proteome are completely conserved, suggesting a loss of specific function or compensatory mutations. To identify functional polymorphisms in conserved Nef sequences, we aligned and analyzed a panel of reference HIV-1 sequences from group M subtypes that were deposited in the NIH Los Alamos HIV Database (http://www.hiv.lanl.gov/content/index). A sequence analysis revealed a subtype C reference strain from Brazil, C.BR92025 (35), that contained three point mutations at otherwise highly conserved residues. These rare point mutations in C.BR92025 were then analyzed for their effect on Nef-mediated MHC-I and CD4 downregulation. Interestingly, we were able to attribute a defect in Nef function to the stability of Nef protein expression, highlighting the importance of these residues in the proper functioning of this key HIV-1 pathogenic factor.

## RESULTS

### Subtype C reference strain C.BR92025 from Brazil has rare point mutations in Nef.

**FIG 1:**
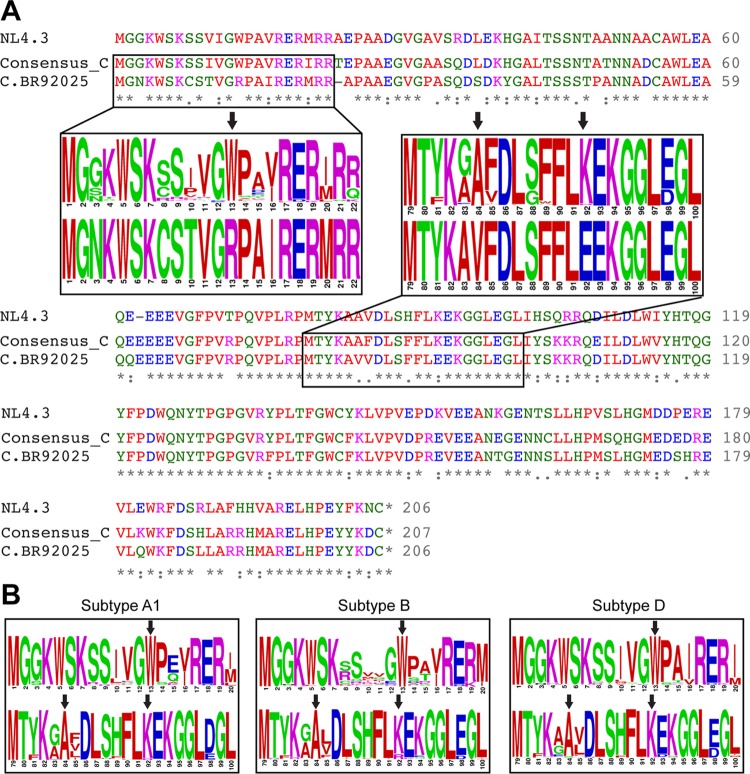
Rare point mutations identified in the Nef protein of subtype C reference strain C.BR92025. (A) Clustal Omega (60, 61) was used to align the Nef protein amino acid sequences of laboratory strain NL4.3, a subtype C consensus sequence from the NIH Los Alamos HIV Database, and subtype C reference strain C.BR92025. Inset boxes show sequence logos with the size of the one-letter amino acid code proportional to the frequency at which that amino acid is found at a given position. Black arrows indicate rare point mutations found in subtype C reference strain C.BR92025. (B) Sequence logos from globally prevalent HIV-1 subtypes (A1, B, and D). Black arrows indicate Nef amino positions 13, 84, and 92; asterisks indicate identical residues; colons indicate conserved residues; and dots indicate semiconserved residues. Blue residues are acidic amino acids, pink residues are basic amino acids, red residues are uncharged nonpolar amino acids, and green residues are uncharged polar amino acids.

The vast genetic diversity of HIV-1 is a major obstacle to the development of an effective vaccine, and the impact of this genetic diversity on the biology of HIV-1 is not completely understood. In order to test the functionality of Nef proteins across various HIV-1 group M subtypes, reference strains from the NIH Los Alamos HIV Database were selected for all subtypes (B, C, D, G, H, J, and K) and subsubtypes (A1, A2, F1, and F2). Nef protein sequence alignments revealed point mutations in the subtype C reference strain (C.BR92025) at positions 13 (W13R), 84 (A84V), and 92 (E92K) that were absent from laboratory strain NL4.3 (Fig. 1A) and from all of the other subtype reference strains analyzed. To determine the prevalence of these point mutations across prominent HIV-1 subtypes, we constructed amino acid sequence logos of Nef-spanning residues 13, 84, and 92 from subtypes A1, B, C and D. These subtypes were selected because of their high global prevalence compared to other group M subtypes. Sequence logos were constructed by selecting up to 10 sequences from each country that had Nef sequences available in the NIH Los Alamos HIV Database (http://www.hiv.lanl.gov/content/index). These sequence logos revealed a high level of conservation of W13, A84, and K92 in other subtype C Nef sequences (Fig. 1A) and across other prominent HIV-1 subtypes (Fig. 1B). When 4,553 full-length Nef protein sequences from the NIH Los Alamos HIV Database were queried, W13 and A84 occurred at a frequency of >99% and K92 occurred at a frequency of 92.75%. However, the frequency of a basic residue at position 92 (K92 or R92) was 99.52%.

### Strain C.BR92025 Nef is poorly expressed at the protein level and has an impaired ability to downregulate CD4 and MHC-I from the cell surface.

**FIG 2:**
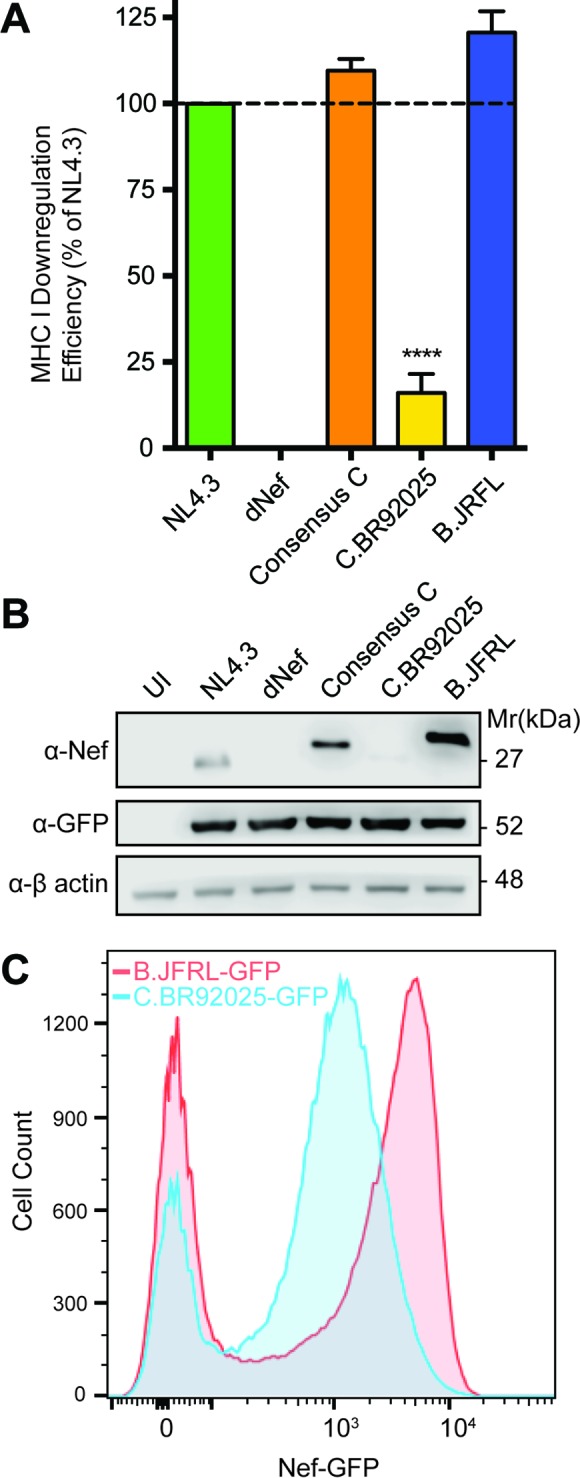
Strain C.BR92025 Nef shows a defect in MHC-I downregulation and protein expression. (A) Jurkat E6.1 cells were infected with lentiviral vectors expressing Nef from laboratory strain NL4.3, lacking Nef expression (dNef), consensus subtype C Nef, or Nef from reference strain B.JRFL or C.BR92025. Cell surface MHC-I levels were measured by flow cytometry at 48 h postinfection with a panspecific anti-MHC-I primary antibody (W6/32) and an Alexa Fluor 647-conjugated secondary antibody. Downregulation efficiency relative to that of NL4.3 was calculated by using MFI (****, *P* < 0.05; *n* = 4). (B) Uninfected (UI) Jurkat E6.1 cells or cells infected with the lentiviral vectors described above expressing nonfusion Nef proteins were collected and lysed at 48 h postinfection. Lysates were analyzed for Nef, EGFP, and actin protein levels by Western blotting. (C) Jurkat E6.1 cells were infected with lentiviral vectors encoding EGFP fused to the Nef proteins of reference strains B.JRFL and C.BR92025. A representative histogram from three independent experiments is shown.

To determine if the W13R, A84V, and K92E mutations identified in subtype C reference strain C.BR92025 impact Nef activity, we measured a key function of HIV-1 Nef, downregulation of the cell surface receptors MHC-I (17) and CD4 (21). To test Nef-mediated MHC-I downregulation, we constructed NL4.3-based replication-deficient lentiviral vectors that express various HIV-1 Nef proteins in the context of isogenic viruses and used these lentiviral vectors to infect the T cell line Jurkat E6.1. Compared to Nef from laboratory strain NL4.3, that from C.BR92025 demonstrated impaired MHC-I downregulation, barely above the levels of a lentiviral vector lacking the Nef protein (dNef), which served as our negative control (Fig. 2A). To ensure that our system was functioning correctly and that the defects in strain C.BR92025 Nef function were not common to HIV-1 subtype C viruses in general, we constructed lentiviral vectors that expressed Nef from a consensus subtype C virus and from a subtype B reference strain, B.JRFL. Jurkat E6.1 cells infected with lentiviral vectors expressing consensus subtype C Nef and strain B.JRFL Nef showed levels of MHC-I downregulation equal to or greater than that of NL4.3 (Fig. 2A), demonstrating that our system can effectively test Nef function and that the observed defects in strain C.BR92025 Nef function are specific to this reference strain.

Interestingly, when Jurkat E6.1 cells infected with lentiviral vectors expressing strain NL4.3 Nef, Nef consensus subtype C, strain C.BR92025 Nef, and strain B.JRFL Nef were analyzed for Nef expression by Western blotting, we were unable to detect high levels of protein in cells infected with the vector encoding strain C.BR92025 Nef. However, vectors encoding strain NL4.3 Nef, consensus subtype C Nef, and strain B.JRFL Nef all expressed Nef at readily detectable levels (Fig. 2B). To independently confirm the reduced expression of strain C.BR92025 Nef, we constructed alternative vectors that encoded strain C.BR92025 Nef and strain B.JRFL Nef fused to the enhanced green fluorescent protein (EGFP). When Jurkat E6.1 cells infected with these vectors were analyzed by flow cytometry, those expressing strain C.BR92025 Nef had a clear reduction in EGFP fluorescence indicative of reduced Nef-EGFP protein levels (Fig. 2C).

**FIG 3:**
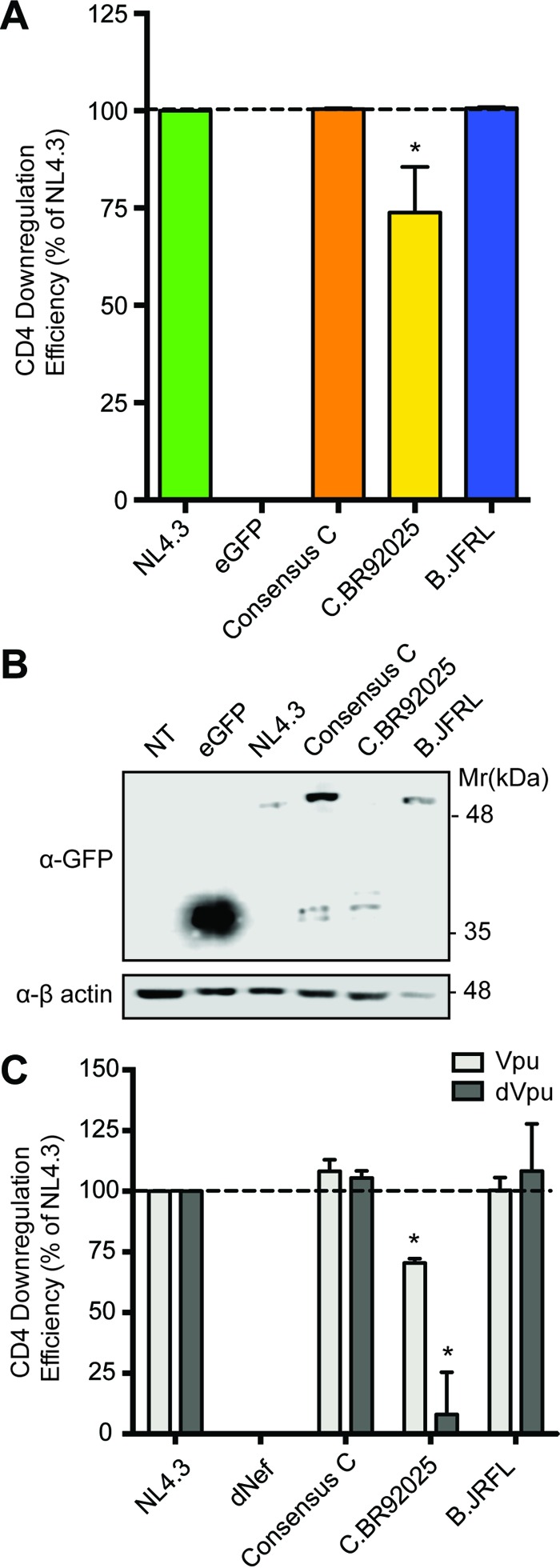
Cells expressing strain C.BR92025 Nef have reduced CD4 downregulation. (A) CD4^+^ HeLa cells were transfected with plasmids encoding EGFP fusion proteins from laboratory strain NL4.3, consensus subtype C Nef, or Nef from reference strain B.JRFL or C.BR92025, as well as nonfused EGFP as a negative control. Cell surface CD4 levels were measured by flow cytometry with an APC-conjugated anti-CD4 antibody at 24 h posttransfection. Downregulation efficiency relative to that of NL4.3 was calculated by using MFI (*, *P* < 0.05; *n* = 3). (B) Lysates of transfected CD4^+^ HeLa cells were collected and lysed at 24 h postinfection. Lysates were analyzed for fusion protein expression by Western blotting with an anti-GFP antibody. (C) Jurkat E6.1 cells were infected with lentiviral vectors either expressing NL4.3 Vpu or not expressing NL4.3 Vpu (dVpu) in addition to expressing Nef of laboratory strain NL4.3, lacking Nef expression (dNef), consensus subtype C Nef, or Nef of reference strain B.JRFL or C.BR92025. Cell surface levels of CD4 were measured by flow cytometry with an APC-conjugated anti-CD4 antibody at 48 h postinfection. Downregulation efficiency relative to that of NL4.3 was calculated by using MFI (*, *P* < 0.05; *n* = 3).

In addition to downregulating MHC-I, HIV-1 Nef can downregulate the cell surface receptor CD4 (21). In order to determine if strain C.BR92025 Nef was also defective in this key function, we analyzed CD4 downregulation in CD4^+^ HeLa cells transfected with plasmids encoding Nef-EGFP fusion proteins with Nef from NL4.3, consensus subtype C, C.BR92025, or B.JRFL. These constructs allowed us to analyze CD4 downregulation independent of HIV-1 Env and Vpu, which can also downregulate CD4 (25). In accordance with our MHC-I downregulation results (Fig. 2A), CD4^+^ HeLa cells transfected with strain C.BR92025 Nef-EGFP showed a significant decrease in CD4 downregulation relative to that of cells transfected with strain NL4.3 (Fig. 3A). This decrease in function was specific to this reference strain, as Nef consensus subtype C-EGFP and strain B.JRFL Nef-EGFP both showed CD4 downregulation equivalent to that of NL4.3 (Fig. 3A). When transfected cells were analyzed for protein expression by Western blotting, we once again observed reduced Nef protein levels in cells expressing strain C.BR92025 Nef-EGFP (Fig. 3B), suggesting that this impaired Nef function is due to poor Nef protein expression.

To investigate Nef-mediated CD4 downregulation in a T cell line and delineate the effects of HIV-1 Vpu, we constructed vectors encoding various Nef proteins that lacked Vpu expression (dVpu). Jurkat E6.1 cells infected with these vectors demonstrated a pattern of CD4 downregulation similar to that in CD4^+^ HeLa cells, with strain C.BR92025 Nef unable to effectively downregulate CD4 (Fig. 3C). Strikingly, this impairment of CD4 downregulation is much more evident when strain C.BR92025 Nef is expressed in the absence of NL4.3 Vpu, suggesting that Vpu partially masks this defect in strain C.BR92025 Nef function. As with the results from the CD4^+^ HeLa cells, vectors encoding Nef consensus subtype C and strain B.JRFL Nef effectively downregulated CD4 in the presence or absence of Vpu (Fig. 3C).

### Decreased expression of strain C.BR92025 Nef is due to rapid protein turnover.

**FIG 4:**
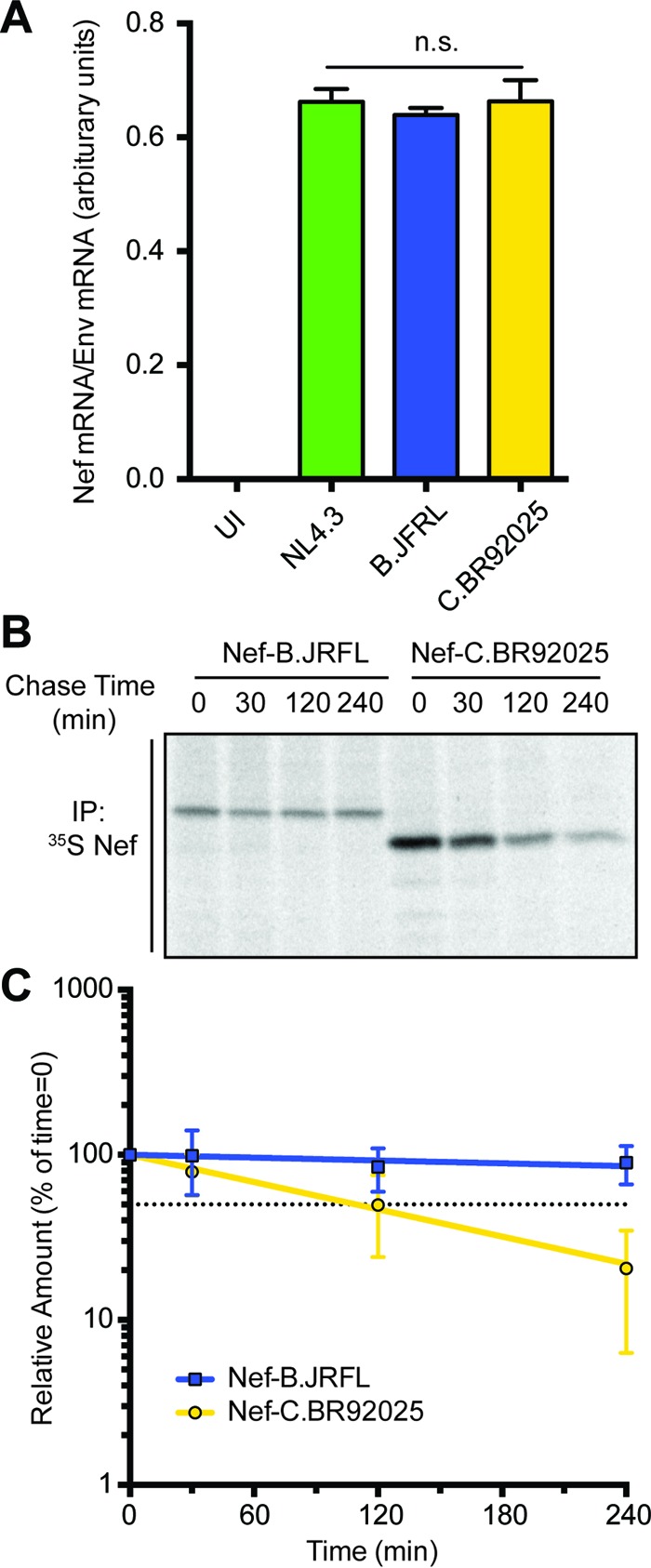
Strain C.BR92025 Nef has an increased rate of protein turnover. (A) Jurkat E6.1 cells were left uninfected (UI) or infected with lentiviral vectors encoding Nef from laboratory strain NL4.3 or reference strain B.JRFL or C.BR92025. At 48 h postinfection, mRNA was isolated from the cells and used for qRT-PCR. Levels of Nef specific mRNA relative to levels of HIV-1 Env-specific mRNA are shown (n.s., not significant; *P* < 0.05; *n* = 2). (B) Jurkat E6.1 cells were infected with lentiviral vectors encoding Nef from reference strain B.JRFL or C.BR92025. At 48 h postinfection, a ^35^S pulse-chase was performed. Cells were lysed and immunoprecipitated with an anti-Nef antibody. Immunoprecipitated (IP) proteins were separated by SDS-PAGE and imaged by autoradiography. A representative image from three independent experiments is shown. (C) The intensities of bands corresponding to immunoprecipitated Nef were determined with ImageQuant 5.2 software and used to calculate the amount of protein remaining relative to the 0-min time point. One-phase nonlinear regression was used to calculate protein half-life. The dotted black line represents 50% of the initial protein remaining (*P* < 0.05; *n* = 3).

Given the vital role that Nef plays in HIV-1 replication and disease progression, the low levels of strain C.BR92025 Nef protein expression were interesting. To further elucidate where these differences in protein expression arise, we investigated Nef mRNA levels by quantitative reverse transcriptase PCR (qRT-PCR) of Jurkat E6.1 cells infected with the lentiviral vectors described above. Interestingly, cells infected with vectors encoding strain NL4.3 Nef, strain C.BR92025 Nef, and strain B.JRFL Nef all displayed similar levels of Nef mRNA, suggesting that the differences in Nef protein expression arise posttranscription (Fig. 4A).

These findings led us to posit that strain C.BR92025 Nef was differentially degraded compared to strain NL4.3 Nef and strain B.JRFL Nef. In order to investigate the rate of Nef protein turnover, we performed a pulse-chase experiment with Jurkat E6.1 cells infected with lentiviral vectors expressing high-expression strain B.JRFL Nef or low-expression strain C.BR92025 Nef. Infected cells were labeled with [^35^S]methionine/cysteine for 30 min (pulse) and then chased in unlabeled medium for up to 240 min. Subsequently, Nef protein was purified by immunoprecipitation with anti-Nef antibody-coated agarose beads, and Nef protein levels were analyzed by autoradiography. Interestingly, the amount of strain C.BR92025 Nef protein remaining after a 240-min chase period was significantly lower than that of strain B.JRFL Nef (Fig. 4B). The data from the pulse-chase experiments were fitted with a one-phase decay nonlinear regression whose slope was used to calculate the half-life of the Nef proteins (Fig. 4C). The regression analysis revealed that strain C.BR92025 Nef was removed from infected cells almost four times as fast as strain B.JRFL Nef (half-life of 2.2 h versus 8.0 h).

### Rare point mutations in strain C.BR92025 Nef affect protein expression and decreased function.

**FIG 5:**
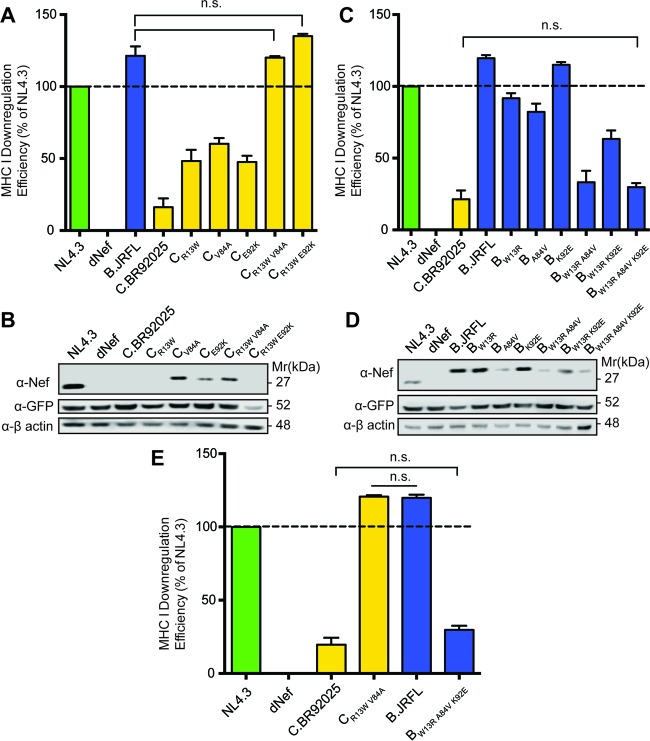
Mutations at residues 13, 84, and 92 are important for the expression and function of HIV-1 Nef. (A) Jurkat E6.1 cells were infected with lentiviral vectors expressing Nef from laboratory strain NL4.3, lacking Nef expression (dNef), or Nef from reference strain C.BR92025, as well as various C.BR92025 mutants. Cell surface MHC-I levels were measured by flow cytometry at 48 h postinfection with a panspecific anti-MHC-I primary antibody (W6/32) and an Alexa Fluor 647-conjugated secondary antibody. Downregulation efficiency relative to that of NL4.3 was calculated by using MFI (n.s., not significant; *P* < 0.05, *n* = 4). (B) Cell lysates from Jurkat E6.1 cells infected with the lentiviral vectors described above were collected and lysed at 48 h postinfection. Lysates were analyzed for Nef, EGFP, and actin protein levels by Western blotting. (C, D) The same experiments described in panels A and B were conducted with lentiviral vectors expressing Nef from reference strain B.JRFL, as well as various B.JRFL mutants. (E) Summary of the results of experiments with lentiviral vectors harboring combination mutant Nef proteins of C.BR92025 (C_R13W V84A_) and B.JRFL (B_W13R A84V K92E_) from panels A and C, respectively.

To determine the role rare point mutations identified in strain C.BR92025 Nef play in the observed decrease in function and protein expression, we carried out a mutational analysis of strain C.BR92025 Nef. Introduction of single point mutations into strain C.BR92025 Nef that revert the amino acids at positions 13, 84, and 92 to the conserved residues normally found in Nef (R13W, V84A, and K92E) each resulted in significant but incomplete rescue of Nef-mediated MHC-I downregulation (Fig. 5A). In addition to MHC-I downregulation, we analyzed the mutants for Nef expression by Western blotting. Interestingly, reversion of position 84 from a valine, as found in strain C.BR92025, to the conserved alanine was sufficient to rescue the expression of strain C.BR92025 Nef despite not fully restoring Nef function (Fig. 5B). To fully restore the function of strain C.BR92025 Nef to the levels of strain NL4.3 Nef, a combination of mutations at position 13 and either position 84 (C_R13W V84A_) or 92 (C_R13W E92K_) was required (Fig. 5A).

In addition to the effects of these rare point mutations on the function of strain C.BR92025 Nef, we were also interested in determining if mutations at residues 13, 84, and 92 were sufficient to disrupt the function and expression of our high-expression subtype B reference, strain B.JRFL Nef. To achieve this, we performed a reciprocal mutational analysis in which we mutated residues 13, 84, and 92 to the amino acids found in strain C.BR92025 Nef, W13R, A84V, and K92E, respectively. Similar to our results with strain C.BR92025 Nef, introduction of single point mutations at positions 13 and 84 only partially disrupted the function of strain B.JRFL Nef; however, mutation at position 92 did not affect the function of strain B.JRFL Nef (Fig. 5C). Interestingly, a single point mutation from alanine to valine at position 84 was sufficient to markedly decrease the amount of Nef protein detected by Western blotting (Fig. 5D), mirroring the results of our mutational analysis of strain C.BR92025 Nef. A combination of mutations at positions 13 and 84 was required to fully disrupt the expression and function of strain B.JRFL Nef. A combination of mutations at positions 13 and 92 only partially disrupted expression and function (Fig. 5C), which is in agreement with our results that showed that the single K92E mutation in strain B.JRFL Nef did not impair its function or expression. These results suggest a critical role for residues W13 and A84 (and, to a lesser extent, E92) in the function of the HIV-1 Nef protein, with residue A84 being responsible for controlling protein expression (Fig. 5E).

### A point mutation at residue 84 targets strain C.BR92025 Nef for rapid removal from cells.

**FIG 6:**
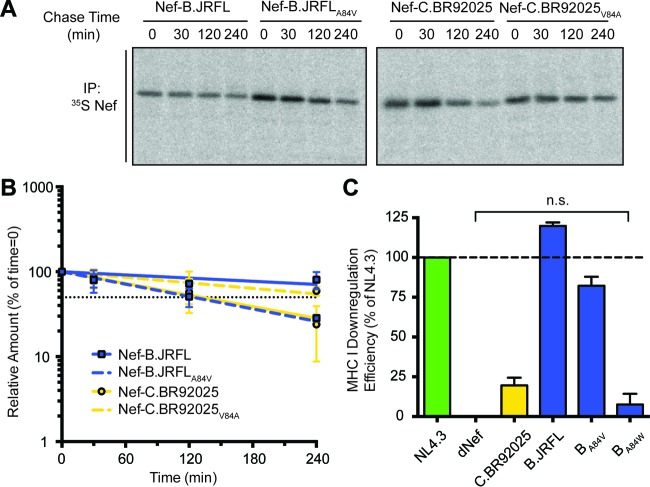
Alanine at position 84 in HIV-1 Nef is critical for sustained expression. (A) Jurkat E6.1 cells were infected with lentiviral vectors encoding Nef of reference strain B.JRFL or C.BR92025, as well as position 84 mutant Nef proteins (B.JRFL_A84V_ and C.BR92025_V84A_). At 48 h postinfection, a ^35^S pulse-chase was performed. Cells were lysed and immunoprecipitated with an anti-Nef antibody. Immunoprecipitated (IP) proteins were separated by SDS-PAGE and imaged by autoradiography. An image representative of three independent experiments is shown. (B) The intensities of bands corresponding to immunoprecipitated Nef were determined with ImageQuant 5.2 software and used to calculate the amount of protein remaining relative to that at the 0-min time point. One-phase nonlinear regression was used to calculate protein half-life. The dotted black line represents 50% of the initial protein remaining (*P* < 0.05; *n* = 3). (C) Jurkat E6.1 cells were infected with lentiviral vectors expressing Nef from laboratory strain NL4.3, lacking Nef expression (dNef), or Nef from reference strain B.JRFL or C.BR92025, as well as residue 84 mutant B.JRFL Nef proteins (B_A84V_ and B_A84W_). Cell surface MHC-I levels were measured by flow cytometry at 48 h postinfection with a panspecific anti-MHC-I primary antibody (W6/32) and an Alexa Fluor 647-conjugated secondary antibody. Downregulation efficiency relative to that of NL4.3 was calculated by using MFI (*P* < 0.05; *n* = 4).

Our mutational analysis suggested that proper and sustained expression of the HIV-1 Nef protein requires an alanine at position 84. To support these findings, we conducted additional pulse-chase experiments with Jurkat E6.1 cells and lentiviral vectors encoding strain B.JRFL Nef and strain C.BR92025 Nef harboring point mutations at position 84. As described above, strain C.BR92025 Nef was removed from cells at a higher rate than strain B.JRFL Nef (Fig. 4). Mutation of the valine at position 84 in strain C.BR92025 Nef to an alanine rescues the protein from rapid removal, more than doubling its half-life (2.2 h to 4.6 h). Similarly, mutation of the alanine at position 84 of strain B.JRFL Nef to a valine is sufficient to dramatically increase the rate at which the protein is removed from cells, shortening its half-life to almost a quarter of that of wild-type strain B.JRFL Nef (8.0 h to 2.1 h) (Fig. 6A and B). The importance of residue 84 is further highlighted by the complete disruption of MHC I downregulation through the introduction of a large bulky tryptophan residue into otherwise highly functional strain B.JRFL Nef (Fig. 6C). These findings suggest that mutating the alanine residue at position 84 in Nef is sufficient to disrupt the expression of HIV-1 Nef.

## DISCUSSION

In the early years of the HIV/AIDS epidemic, the viral isolates used for research studies were predominantly from group M subtype B because of the very high prevalence of subtype B in North America and Western Europe, where much of the early research was taking place (33, 34). As the extensive genetic diversity of HIV-1 became apparent, there was a need to develop a panel of full-length reference strains for the various HIV-1 subtypes (36). Reference strain C.BR29025 was isolated from a 23-year-old male hemophilia patient in Brazil in 1992 and later deposited into the NIH Los Alamos HIV Database (35). Upon phylogenic analysis, full-length C.BR92025 clustered with other subtype C reference strains, suggesting that it was not dramatically different from other subtype C viruses (35–37). These reference strains were selected to allow for intersubtype comparison of HIV-1 proteins, specifically, the less-well-studied regulatory proteins; however, they were selected on the basis of the availability of full-length sequences without verification that all of the proteins functioned properly (36).

The clinical progression of this patient is not well documented, but at the time of sampling, he had active viral replication with detectable viral titers and p24 levels (35). In addition, C.BR92025 has been used in studies as a reference strain and is replication competent *in vitro* (38, 39). However, given the importance of HIV-1 Nef for disease progression, decreased expression of this key accessory protein should result in decreased viral replication. Indeed, when C.BR92025 has been used as a reference strain, it has demonstrated less fitness than HIV-1 laboratory strain BaL (39). Interestingly, in competition assays to determine viral fitness, C.BR92025 was outcompeted by viral isolates from long-term survivors (39). These findings demonstrate that C.BR92025 has a decreased replicative capacity, but the authors did not explore these differences further, so it is not possible to attribute defects in replication directly to Nef. Analysis of Nef sequences from a separate cohort comparing elite controllers with chronic progressors showed no increase in the prevalence of a mutation at residue 13, 84, or 92 between the two groups (40). However, as this cohort was limited to 45 viruses from each group, a much larger database is needed to draw definitive conclusions about the role of these mutations in disease progression.

Given the high mutation rate of HIV-1 and the strong immunogenicity of the Nef protein (41), highly conserved residues suggest functional and/or structural importance. As a result, we were intrigued to discover that subtype C reference strain C.BR92025 contained three rare residues at positions 13, 84, and 92 (NL4.3 numbering). Across all of the group M subtypes found in the NIH Los Alamos HIV Database, these residues exist almost exclusively as a tryptophan (13), an alanine (A84), and a basic amino acid (K/R92), whereas in subtype C reference strain C.BR92025, these residues are an arginine (R13), a valine (V84), and a glutamic acid (E92) (Fig. 1A). To explore the effect of these mutations on Nef function, we focused on Nef-mediated MHC-I and CD4 downregulation, two well-documented Nef functions (17–21). The downregulation of both MHC-I and CD4 was significantly impaired in strain C.BR92025 Nef compared to that in strain NL4.3 Nef and Nef proteins from a consensus subtype C virus and subtype B reference strain B.JRFL (Fig. 2A and 3A). Furthermore, these impairments corresponded to a decrease in Nef protein levels in infected or transfected cells (Fig. 2B and 3B). These findings were of interest because the affected residues lie outside the well-described motifs in Nef known to be required for proper receptor downregulation. Of the three residues, only W13 has been implicated in MHC-I downregulation (42). The importance of CD4 downregulation for HIV-1 replication is highlighted by the redundant functions of the viral proteins Nef, Vpu, and Env, all of which can decrease CD4 levels on the infected cell surface (16, 25). This functional redundancy may have been enough to compensate for the defects in Nef that we have identified and allow this strain to establish an initial infection despite its decreased replication efficiency (39).

Minimal literature exists on naturally occurring residues altering the expression of HIV-1 Nef; therefore, we decided to explore low-expression strain C.BR92025 Nef further. qRT-PCR analysis revealed equivalent mRNA levels, suggesting that the differences in protein levels were arising posttranscription. In agreement with these findings, strain C.BR92025 Nef has a half-life that is a little over a quarter of that of high-expression strain B.JRFL Nef (2.2 h versus 8.0 h) (Fig. 4C). This high rate of protein turnover explains why strain C.BR92025 Nef is barely detectable by Western blotting despite mRNA levels equal to those of strain B.JRFL Nef. Interestingly, at the 0-min chase time point, the intensity of the band corresponding to strain C.BR92025 Nef exceeds that of strain B.JRFL Nef. This increased intensity may be due to the presence of two additional sulfur-containing methionine residues in strain C.BR92025 Nef relative to strain B.JRFL Nef, allowing for increased incorporation of ^35^S. Regardless, this time point should capture all of the newly synthesized protein produced during the 30-min pulse, with no time for protein turnover, suggesting that strain C.BR92025 Nef is being continually produced at a high level but is removed from cells very efficiently. This efficient removal of Nef from infected cells results in increased surface MHC-I and CD4 levels.

To confirm the role of the rare point mutations in the observed decreases in Nef function and protein levels, we conducted a mutational study with both subtype B and C reference strains B.JRFL and C.BR92025, respectively. Although our findings were not completely complementary between high-expression strain B.JRFL Nef and low-expression strain C.BR92025 Nef, both sets of experiments found an important role for W13 and A84 in the expression and function of HIV-1 Nef. Specifically, W13 appears to be required for efficient MHC-I downregulation, as a W13R mutation significantly reduces the MHC-I downregulation efficiency of strain B.JRFL Nef, whereas an R13W mutation rescues the MHC-I downregulation efficiency of strain C.BR92025 Nef. It is important to note that the role of W13 appears to be independent of protein expression levels, as these mutations do not alter the detection of the respective proteins by Western blotting (Fig. 5B and D). However, an A84V mutation was sufficient to markedly decrease Nef protein levels in high-expression strain B.JRFL Nef, while a V84A mutation was sufficient to restore Nef to detectable levels in low-expression strain C.BR92025 Nef. These changes in protein levels were accompanied by corresponding changes in MHC-I downregulation, suggesting that altered protein levels played a role in the impairment of Nef function. The role of the third rare point mutation, E92, is less clear. Our mutational studies of strain C.BR92025 Nef suggest an important role for K92 in the function and expression of Nef, as the restorative E92K mutation partially rescues the function and expression of strain C.BR92025 Nef. However, in the context of strain B.JRFL Nef, the K92E mutation does not significantly impact function and expression.

**FIG 7:**
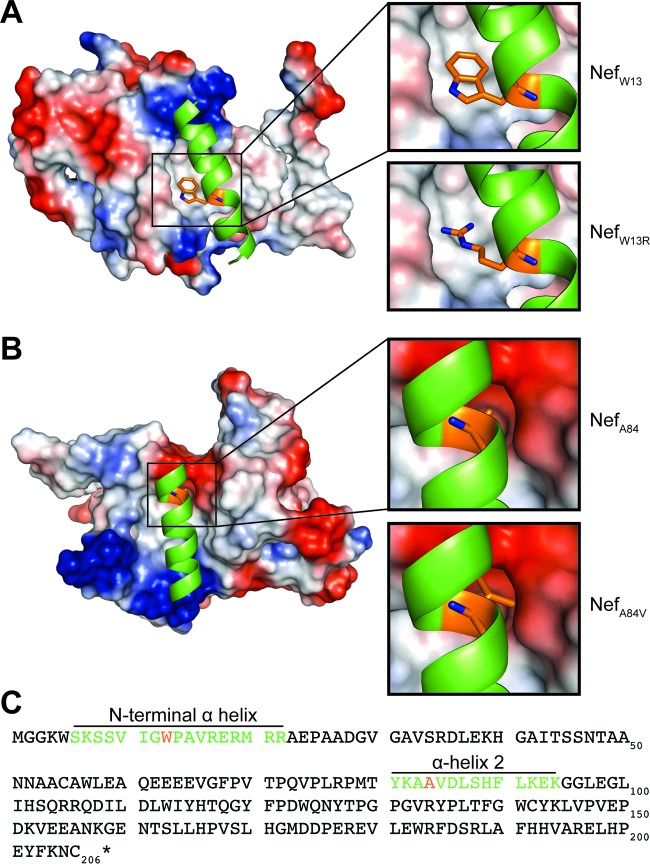
Potential intramolecular interactions of Nef W13 and A84. Structural model of HIV-1 Nef (PDB ID 4EN2). (A) The N-terminal alpha helix of HIV-1 Nef depicted as a cartoon structure in green with the side chain of W13 in orange. The remaining Nef structure is shown by surface representation with electrostatic potentials in red (negative charge) and blue (positive charge). Zoomed-in images showing W13 (top right) and mutant W13R (bottom right) are also shown. (B) Nef alpha helix 2 depicted as a cartoon structure in green with the side chain of A84 in orange. The remaining Nef structure is shown by surface representation with electrostatic potentials in red (negative charge) and blue (positive charge). Zoomed-in images showing A84 (top right) and mutant A84V (bottom right) are also shown. (C) Amino acid sequence of NL4.3 Nef with the N-terminal alpha helix and alpha helix 2 depicted with the colors described in panels A and B.

Our findings that W13 plays an important role in MHC-I downregulation are in agreement with a previous structural study of Nef in complex with MHC-I and AP-1 (42). This model suggests an intramolecular interaction of W13, located in the N-terminal alpha helix, with a hydrophobic pocket in the core of Nef (Fig. 7A). This docking of the N-terminal helix is thought to be required for proper positioning of Nef at membranes, allowing for the formation of the Nef, MHC-I, and AP-I complex. This was supported by a W13A mutation that disrupted MHC-I downregulation and the ability of Nef to pull down AP-1 *in vitro* (42). The presence of the charged arginine residue at position 13 in strain C.BR92025 Nef would presumably disrupt this intramolecular interaction (Fig. 7A), explaining the observed decrease in MHC-I downregulation.

To our knowledge, this is the first report of a role for A84 in the proper expression of HIV-1 Nef and therefore we were interested in determining if the altered rate of protein turnover we observed was attributable to this residue. Pulse-chase analysis of cells infected with lentiviral vectors encoding strain B.JRFL Nef, C.BR92025 Nef, and the corresponding position 84 mutant proteins revealed that these single-amino-acid mutations were sufficient to alter the rate of protein turnover. When an A84V mutation was introduced into strain B.JRFL Nef, its pulse-chase profile shifted to resemble that of strain C.BR92025 Nef, whereas a V84A mutation introduced into strain C.BR92025 Nef shifted its pulse-chase profile to resemble that of strain B.JRFL Nef (Fig. 6A and B).

These findings were particularly interesting, as alanine and valine are quite similar in structure, with their side chains differing by just two methyl groups. Analysis of previously reported structural models of HIV-1 Nef indicate that residue 84 is located within alpha helix 2 and is oriented toward the protein core (Fig. 7B) (42). Therefore, the increased size of the valine side chain may cause intramolecular steric hindrances that could affect the structure of strain C.BR92025 Nef. This hypothesis is supported by a tryptophan mutation (A84W) that introduces a large bulky residue and more severely disrupts Nef receptor downregulation (Fig. 6C). Accumulated strain C.BR92025 Nef could be targeted for removal from the cell by various protein degradation pathways (43, 44), resulting in the observed increase in protein turnover.

The conflicting results of the mutational studies in regard to residue 92 preclude conclusions on its role in Nef function. The finding of our strain B.JRFL Nef mutational studies that residue 92 did not affect MHC-I downregulation is in agreement with a recent report that mutation of the KEK94 motif failed to disrupt MHC-I downregulation (45). In addition, an earlier study demonstrated that K92 was dispensable for Nef-mediated CD4 downregulation (46), further suggesting a limited role in Nef function.

Interestingly, there are a number of MHC-I epitopes that span residue 84, suggesting strong immune pressure against consensus protein sequences in that region (41, 47–49). Despite increased immune pressure, the presence of an alanine at position 84 appears to be under positive selection, further supporting its importance in the function and/or expression of Nef (47, 48). This study adds to other protein-wide screens of polymorphisms to assess the genetic robustness of HIV-1 proteins such as integrase (50) and capsid (51). Identification of highly conserved residues that appear to have strict structural constraints may be useful for the development of T-cell-based vaccines that include Nef as a target for CTL responses.

In conclusion, we have identified a subtype C reference strain deposited in the NIH Los Alamos HIV Database that contains three point mutations at otherwise highly conserved residues in HIV-1 Nef. We have shown that the presence of these point mutations is responsible for the decreased function and expression of the viral protein. Of note, an alanine at residue 84, located in Nef alpha helix 2, appears to be essential for the proper expression of HIV-1 Nef. The importance of residue 84 in Nef biology provides an additional molecular target for disrupting Nef function, an anti-HIV-1 approach that has gained interest in both the cure and treatment fields (52, 53).

## MATERIALS AND METHODS

### Cell culture.

CD4^+^ HeLa (American Type Culture Collection, Manassas, VA) and HEK 293T cells (Life Technologies, Carlsbad, CA) were grown in complete Dulbecco’s modified Eagle’s medium (DMEM) containing 10% fetal bovine serum (FBS; Wisent, Quebec, Canada) and 100 µg/ml penicillin/streptomycin (HyClone, Logan, UT). Jurkat E6.1 T cells (catalog no. 177; National Institutes of Health AIDS Research and Reference Reagent Program) were cultured in RPMI 1640 supplemented as described above with the addition of 1% sodium pyruvate, 1% nonessential amino acids, and 2 mM l-glutamine (HyClone). All cell lines were grown at 37°C in the presence of 5% CO_2_ and subcultured in accordance with the supplier’s recommendations.

### Proviral plasmids and cloning strategy.

The pNL4-3 Δ*gag*/*pol* EGFP replication-incompetent HIV-1 proviral vector (54, 55) was used as the base template for modification of the viral expression vector system. Primer overlap extension mutagenesis (56) was used to amplify two fragments flanking the Nef coding sequence in order to remove Nef and insert XmaI and NotI restriction sites, as described previously (55). Various Nef coding sequences were amplified from expression plasmids generously provided by Thomas Smithgall (University of Pittsburgh School of Medicine) or synthetically generated with Invitrogen GeneArt Synthesis (Invitrogen). Subtype-specific primers were used to introduced XmaI and NotI sites at the 5′ and 3′ ends of Nef, respectively, enabling the insertion of Nef sequences into the pNL4-3 Δ*gag*/*pol* EGFP vector.

Nef-EGFP fusion protein plasmids were generated by inserting various Nef sequences into the pN1-EGFP expression plasmid (TaKaRa, Mountain View, CA). pN1-EGFP was digested with AgeI, and NotI and Nef coding sequences were introduced with subtype-specific primers that added AgeI and NotI sites to the 5′ and 3′ ends of Nef, respectively.

Site-directed mutagenesis (57) was performed to generate point mutations in Nef. Mutagenic primers were designed with Agilent Technologies Primer Design software, and Agilent Technologies protocols (58) were used to introduce point mutations. Sequencing performed at the University of Western Ontario Genomics Center confirmed all point mutations.

### Lentiviral production and processing.

Lentiviral vectors were produced in HEK 293T cells. Cells were triple transfected with PolyJet (FroggaBio, Toronto, ON, Canada) and pNL4-3 Δ*gag*/*pol* EGFP, strain NL4.3 Nef, or the modified plasmids, as well as pdR8.2 and pMD2.G (pdR8.2 and pMD2.G were provided by Didier Trono [Addgene plasmids 12263 and 12259, respectively]) as previously described (59). Lentiviral vectors were harvested at 48 h posttransfection. Briefly, virus-containing medium was centrifuged at 3,000 × *g* for 5 min to remove cellular debris and subsequently passed through a 0.2-µm filter. Filtered supernatant was supplemented with an additional 20% FBS prior to storage at −80°C.

### Flow cytometry.

To quantify cell surface MHC-I expression levels, Jurkat E6.1 cells were infected with the viruses indicated and fixed in 2% paraformaldehyde (PFA) 48 h later. Cells were surface stained for MHC-I with W6/32 (anti-MHC-I, panselective, provided by D. Johnson, Oregon Health and Sciences University). Background fluorescence was verified with a no-primary-antibody control, which revealed a nil value. Cell surface MHC-I expression was quantified by flow cytometry (BD FACSCanto II), and the data were analyzed with FlowJo software (version 9.6.4; TreeStar, Ashland, OR). Infected cells were first gated by selecting for EGFP-positive cells, and then MHC-I downregulation efficiency was calculated with the formula MHC-I downregulation efficiency (% of NL4.3) = (MFI_exp_ − MFI_dNef_/MFI_NL4.3_ − MFI_edNef_) × 100%.

MHC-I downregulation efficiency was reported as a percentage of that of NL4.3, where 100% represents MHC-I downregulation efficiency equivalent to that of NL4.3 and 0% is equivalent to infection with a virus lacking Nef (dNef). MFI_exp_ represents the mean fluorescence intensity (MFI) of surface MHC-I on cells infected with our experimental samples, MFI_dNef_ represents surface MHC-I MFI on cells infected with a virus that does not express Nef, and MFI_NL4.3_ represents the MFI of cells infected with our virus expressing NL4.3 Nef. As a reference, NL4.3 Nef removed a median of 62% ± 7% of the MHC-I removed from the cell surface by dNef.

Cell surface CD4 on Jurkat E6.1 cells was detected with the same protocol and an allophycocyanin (APC)-conjugated anti-CD4 monoclonal antibody (clone OKT4; BioLegend, San Diego, CA). As a reference, NL4.3 Nef removed a median of 51% ± 9% of the CD4 removed from the cell surface by dVpu dNef.

To quantify the CD4 on the surface of CD4^+^ HeLa cells, cells were collected 24 h posttransfection by washing with phosphate-buffered saline and trypsinization, followed by fixation in 2% PFA. Fixed cells were stained with APC-conjugated OKT4 and analyzed by flow cytometry (BD FACSCanto II) and the FlowJo software. Transfected cells were first gated on EGFP-positive cells, and then CD4 downregulation efficiency was calculated with the formula CD4 downregulation efficiency (% of NL4.3) = (MFI_exp_ − MFI_EGFP_/MFI_NL4.3_ − MFI_EGFP_) × 100%.

CD4 downregulation efficiency was reported as a percentage of that of NL4.3, where 100% represents CD4 downregulation efficiency equivalent to that of NL4.3 and 0% is equivalent to transfection with a plasmid expressing EGFP alone. MFI_exp_ represents the MFI of surface CD4 on cells transfected with our experimental samples. MFI_EGFP_ represents the MFI of surface CD4 on cells transfected with a plasmid expressing EGFP alone. MFI_NL4.3_ represents the MFI of cells transfected with a plasmid expressing strain NL4.3 Nef-EGFP. As a reference, NL4.3 Nef-EGFP removed 89% ± 2% of the CD4 removed from the cell surface by EGFP alone.

### Western blot assays.

Infected Jurkat E6.1 cells or transfected HeLa cells were collected at 48 or 24 h, respectively, and lysed in lysis buffer (0.5 M HEPES, 1.25 M NaCl, 1 M MgCl_2_, 0.25 M EDTA, 0.1% Triton X-100, and 1× Complete protease inhibitor tablets [Roche, Indianapolis, IN]). Cells were lysed at 4°C while rotating for 20 min before insoluble cellular debris was removed by centrifugation at 20,000 × *g* for 20 min. Lysates were boiled at 98°C in 5× SDS-PAGE sample buffer (0.312 M Tris [pH 6.8], 25% 2-mercaptoethanol, 50% glycerol, 10% SDS), and proteins were separated by 12% SDS-PAGE and subsequently transferred to nitrocellulose membranes. The membranes were blocked in 5% nonfat skim milk (BioShop, Burlington, ON, Canada) in TBST (Tris-buffered saline, 0.1% Tween 20) containing 0.1% Triton X-100 for 45 min and then incubated overnight at 4°C with various primary antibodies, i.e., rabbit anti-Nef polyclonal antibody (1:2,500; catalog no. 2949; NIH AIDS Research and Reference Reagent Program, United States), rabbit anti-GFP polyclonal antibody (1:3,000; Clontech, TaKaRa), or mouse anti-β-actin monoclonal IgG (1:3,000; Thermo Scientific). Membranes were washed three times in TBST containing 0.1% Triton X-100 and incubated for 2 h at room temperature with the appropriate species-specific horseradish peroxidase-conjugated antibodies (1:3,000; Thermo Scientific). Blots were developed with ECL substrates (Millipore Inc., Billerica, MA) and a C-DiGit chemiluminescence Western blot scanner (LI-COR Biosciences, Lincoln, NE).

### qRT-PCR.

Jurkat E6.1 cells were infected with various lentiviral vectors at equivalent infection rates based on EGFP fluorescence. RNA was collected from infected Jurkat E6.1 cells with the PureLink RNA minikit (Invitrogen) at 48 h postinfection and stored at −80°C. Purified RNA was reverse transcribed into bulk cDNA with the SuperScript III First-Strand Synthesis System. cDNA was used as a template for qRT-PCR with the SensiFAST SYBR No-ROX kit (FroggaBio, Toronto, ON, Canada) to amplify Env- and Nef-specific amplicons with the following primers: common Env and Nef fwd 5′-GGCGGCGACTGAAGAAG, Env rev 5′-ACTATGGACCACACAACTATTGCT, and Nef rev 5′-GATTGGGAGGtGGGTTGCT. qRT-PCR runs were performed with a Rotor-Gene 6000 (Qiagen, Valencia, CA) under the following conditions: 1 cycle of 95°C for 2 min for polymerase activation and 40 cycles of 95°C for 5 s for denaturation, 60°C for 10 s for annealing, and 72°C for 15 s for extension. Relative levels of Nef and Env mRNAs were calculated from standard curves generated from plasmids carrying the respective genes at known concentrations.

### Pulse-chase experiment.

Jurkat E6.1 cells were infected with specific lentiviruses for 48 h postinfection and subsequently incubated in starvation medium (DMEM lacking l-cysteine and l-methionine supplemented with 5% Gibco Dialyzed FBS [Thermo Fisher] and Gibco GlutaMAX) for 30 min. The starvation medium was removed, and the cells were pulsed with pulse medium (starvation medium supplemented with EXPRESS^35^S Protein Labeling Mix [^35^S] [PerkinElmer, Guelph, ON, Canada] at 55 µCi/ml) for 30 min. Cells were washed with complete RPMI supplemented with 20% FBS and then chased with the same medium for 0, 30, 120, or 240 min at 37°C. After the chase period, cells were lysed in 800 µl of RIPA-Doc buffer (140 mM NaCl, 10 mM NaH_2_PO_4_, 1% GEPAL CA-630, 0.05% SDS, 0.5% sodium deoxycholate) by rocking at 4°C for 10 min, followed by centrifugation at 20,000 × *g* at 4°C for 10 min. Following centrifugation, the supernatant was removed and immunoprecipitated by incubation with anti-Nef antibody (catalog no. 2949; NIH AIDS Reagent Program)-conjugated protein A agarose beads (Sigma-Aldrich, St. Louis, MO) overnight at 4°C while rocking. The agarose beads were then washed twice with 1 ml of RIPA-Doc and resuspended in 20 µl of 5× SDS loading buffer prior to being boiled for 5 to 10 min at 95°C. Proteins were separated by SDS-PAGE on a 12% gel and then fixed (50% methanol and 10% acetic acid in water) for 5 to 10 min. Gels were dried for 2 h on Whatman paper with a model 583 Gel Dryer (Bio-Rad, Hercules, CA), and the autoradiography signal was captured by placing the dried gel in a phosphor storage cassette for 48 h and developing it on a Storm 820 phosphorimager (GE Healthcare, Chicago, IL). ImageQuant 5.2 (GE Healthcare) software was used to quantify band intensity.

### Sequence logo generation.

Sequence logos were generated with WebLogo software (http://weblogo.berkeley.edu/logo.cgi) by using input sequences collected from the NIH Los Alamos HIV Database. Sequences were selected from each country that contained a given subtype, with a maximum of 10 sequences selected from each country. Sequences were then screened for any large deletions or incomplete sequences. Residues in the sequence logos were colored on the basis of Clustal Omega (60, 61) sequence alignments, where blue residues are acidic amino acids, pink residues are basic amino acids, red residues are uncharged nonpolar amino acids, and green residues are uncharged polar amino acids. Frequencies of polymorphisms at positions 13, 84, and 92 were determined with the AnalyzeAlign tool found at the NIH Los Alamos HIV Database to query 4,553 full-length Nef protein sequences selected with the Filtered Web Search (http://www.hiv.lanl.gov/content/index).

### Structural modeling.

Mac PyMOL 3 software (Schrödinger, New York, NY) was used to construct structural models depicting Nef residues W13 and A84 (PDB ID 4EN2) (42).

### Statistical analysis.

Statistical analysis was conducted with GraphPad Prism software (GraphPad Software, Inc., La Jolla, CA). Downregulation and qRT-PCR data were analyzed by one-way analysis of variance, while pulse-chase data were analyzed by one-phase decay nonlinear regression.
